# Surgical options in oroantral fistula management: a narrative review

**DOI:** 10.1186/s40729-018-0152-4

**Published:** 2018-12-27

**Authors:** Puria Parvini, Karina Obreja, Robert Sader, Jürgen Becker, Frank Schwarz, Loutfi Salti

**Affiliations:** 1 Department of Oral Surgery and Implantology, Carolinum, Johann Wolfgang Goethe-University, Frankfurt, Germany; 2 Department for Oral, Cranio-Maxillofacial and Facial Plastic Surgery, Medical Center of the Goethe University Frankfurt, Frankfurt am Main, Germany; 3 Department of Oral Surgery, Universitätsklinikum Düsseldorf, Düsseldorf, Germany

**Keywords:** Oroantral, Fistula, Flaps, Grafts, Maxillary sinus, Complication management, Oral surgery

## Abstract

An oroantral fistula (OAF) is a pathological abnormal communication between the oral cavity and the maxillary sinus which may arise as a result of failure of primary healing of an OAF, dental infections, osteomyelitis, radiation therapy, trauma, or iatrogenic complications. With the presence of a fistula, the maxillary sinus is permanently open. Microbial flora passes from the oral cavity into the maxillary sinus, and the inflammation of the sinus occurs with all potential consequences. In literature, various techniques have been proposed for closure of OAFs. Due to the heterogeneity of the data and techniques found, we opted for a narrative review to highlight the variety of *techniques* discussed in the literature. Techniques *of particular interest* include the bone sandwich with resorbable guided tissue regeneration (GTR) membrane and platelet-rich fibrin (PRF) used alone as both a clot and a membrane. The great *advantage of these techniques* is that no donor site surgery is necessary, making the outcome valuable in terms of time savings, cost and, more importantly, less discomfort to the patient. Additionally, both bony and soft tissue closure is performed for OAF, in contrast to flaps, which are typically used for procedures in the sinus area. The reconstructed bony tissue regenerated from these techniques will also be appropriate for endosseous dental implantation.

## Background

An oroantral fistula (OAF) can be defined as an epithelialized pathological unnatural communication between the oral cavity and the maxillary sinus [1]. The term oroantral fistula is used to indicate a canal lined by epithelium that may be filled with granulation tissue or polyposis of the sinus membrane [2]. They can arise as late sequelae from perforation and last at least 48–72 h. An oroantral fistula (OAF) may develop as a complication of maxillary molar or premolar extraction due to the proximity of the bicuspid apices and molars to the antrum [3]. Furthermore, oroantral fistula might originate following the removal of maxillary cysts or tumors, facial trauma, dentoalveolar or implant surgery, and infection or may even be iatrogenic in nature. Oroantral fistulas are common between the ages of 30 and 60 [1]. Apparently, studies reported sexual dimorphism in oroantral fistula, with males showing more frequency than females [1, 4]. This difference can be explained by a higher overall frequency of traumatic tooth extraction in men [5].

Clinically, the patient may experience one or more disturbances which draw attention to the oroantral fistula. Symptoms and signs comprise, pain, foul or salty taste, alteration in voice resonance, inability to blow out the cheeks, air shooting from the fistula into the mouth when blowing the nose, and escape of liquids from the mouth through the nose [5].

At a later stage, the formation of an antral polyp, which is visible through the defect intraorally, is possible. The establishment of oroantral communication can be confirmed by the Valsalva method. The patient is instructed to expel air against closed nostrils, while the clinician checks if air hisses from the fistula into the mouth. A hissing noise from air leakage through the maxillary sinus and nose indicates a positive test. In some cases, the test of blowing through the nose or mouth does not provide a positive answer, particularly when the fistular canal is filled with inflammatory changed nasal mucous membrane. Additionally, a test with a blunt probe will confirm the existence of an oroantral fistular canal. However, to confirm clinical findings, the clinician needs to radiologically inspect the site via a panoramic radiograph or a computed tomography (CT).

Radiologically, in the computed tomography (CT) or cone beam computed tomography (CBCT), the oroantral fistula might show as sinus floor discontinuity, opacification of the sinus, or communication between the oral cavity and the sinus. In addition, focal alveolar atrophy and associated periodontal disease may be observed [6]. In chronic OAF, there is generalized mucosal thickening. Recent studies revealed that an oroantral fistula should be closed within 24 h. After this period, the inflammation of the sinus through contamination of the oral cavity makes it impossible to effectively conduct the treatment [7, 8]. Guven reported that sinus infection can occur with any size and duration of the fistula canal [3]. Accordingly, symptoms associated with inflammation in the sinus should be cured medically with antibiotics before closure of an (OAF) to avoid impaired drainage. Without *treatment*, *fistula* often leads to chronic OAFs, which usually lead to severe chronic inflammatory thickening of the sinus membrane. Closure of the defect aims to prevents oral bacteria and food debris from penetrating the sinus. According to different authors, small fistulae tend to heal spontaneously, whereas larger fistulae rarely heal [3]. Surgery is indicated if a fistula does not heal within 3 weeks [2, 3, 9]. The aim of surgery is to remove the diseased bone and to resect the thickened epithelium along the borders of the fistula [3, 9].

Historically, several methods of OAF closure have been reported in the literature. However, none of these methods are proved to be superior to the others. Additionally, each method presents certain advantages and disadvantages. The goal of this literature is to provide a review of the surgical treatment strategies of OAFs, including their advantages and disadvantages.

## Materials and methods

**Fig. 1:**
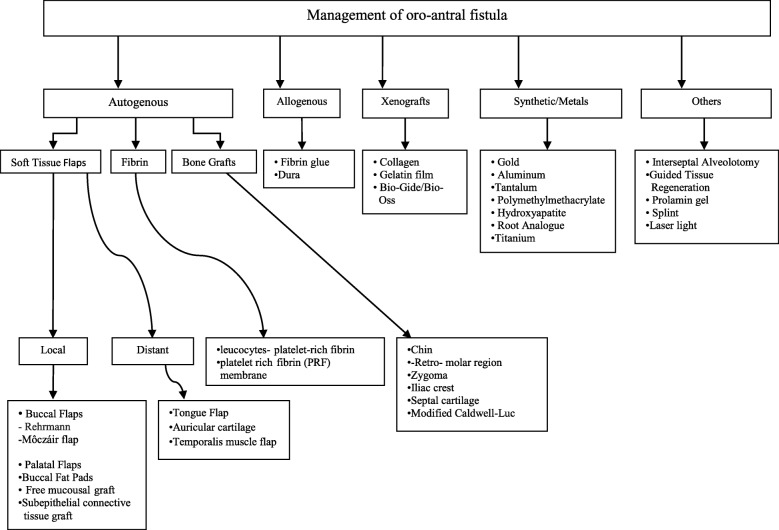
Treatment strategies for OAF closure

A narrative literature review of articles and case reports for oroantral fistula has been conducted in the PubMed databases of published English literature. Articles published until April 2018 were reviewed. In addition to 262 articles on the closure of oroantral, 4 articles on the closure of antrooral fistula in humans, and 5 articles in animals, citations were referenced to identify further relevant articles. According to Visscher’s classification, the treatment strategies for OAFs closure can be broadly categorized into autogenous soft tissue grafts, autogenous bone grafts, allogenous materials, xenografts, synthetic closure, and other techniques [10] (Fig. 1). New techniques were included in this classification. The studies and number of cases are listed in Table 1.

**Table 1:** Studies on surgical techniques for closure OAFs

Author year	No. of participants	Method
Autogenous soft tissue flaps		
Lin et al. 1991	16	Buccal flap
Ehrl 1980	175	Palatal flaps
Anavi et al. 2003	63	Palatal mucoperiosteal rotation flap
Choukas 1974	1	Palatal straight advancement flap
Hynes 1955	?	Palatal hinged flap
Henderson 1974	1	Palatal pedicled island flap
Salins et al. 1996	12	Palatal anteriorly based flap
Dergin et al. 2007	?	Modified submucosal connective tissue flap
Ito and Hara 1980	13	Submucosal connective tissue pedicle flap
Yamazaki et al. 1985	7	Submucosal island flap
Marcantonio C et al. 2015	1	Palatal pedicle flap
Lee et al. 2002	21	Random palatal flap
Guerrero-Santos et al. 1966	10	Tongue flap
Pourdanesh et al. 2013	1	Temporalis muscle flap
Autogenous bone grafts		
Proctor 1969	18	Iliac crest
Haas et al. 2003	3	Chin bone grafts
Watzak et al. 2005	4	Retromolar bone grafts
Peñarrocha-Diago et al. 2007	1	Zygomatic bone
Kapustecki et al. 2016	20	Autogenous bone graft and platelet-rich fibrin
Allogenous		
Gattinger 1984	23	Fibrin glue in combination with a collagen sheet
Stajcic 1985	16	Fibrin glue
Frenkel 1990	29	Lyophilized dura
Aladag et al. 2018	24	Modified Caldwell-Luc
George 2018	1	Leucocytes- platelet-rich fibrin
Assad et al. 2018	2	Platelet-rich fibrin (PRF) clot and (PRF) membrane
Xenografts		
Mitchell 1983	30	Lyophilized porcine dermis
Shaker et al. 1995	40	Lyophilized porcine dermis (Zenoderm)
Ogunsalu 2005	1	Bio-Guide (porcine collagen membrane) and Bio-Oss (bovine bone grafting material)
Synthetic/metals closure		
McClung and Chipps 1951	4	Tantalum foil
Goldman 1969	1	Gold foil technique
Al Sibahi and Shanoon 1982	10	Self-curing polymethylmethacrylate
Becker et al. 1987	20	Dense hydroxylapatite
Becker et al. 1987	20	Hydroxylapatite implants
Thoma et al. 2006	20	Root analog made of β -tricalcium phosphate
Steiner et al. 2008	8	36-gauge pure aluminum plates
Procacci et al. 2016	12	Functional endoscopic sinus surgery and titanium mesh
Other techniques		
Götzfried and Kaduk 1985	Animal study	Prolamin gel
Waldrop and Semba 1993	2	Guided tissue regeneration
Hori et al. 1995	8	Interseptal alveolotomy
Grzesiak-Janas and Janas 2001	61	Laser light
Logan et al. 2003	1	Palatal splint
Kitagawa et al. 2003	2	Third molar transplantation

### Autogenous

Closure of OAF can be achieved using different flap techniques, each of which presents both advantages and limitations. Three types of flaps are most widely used: a buccal flap, a buccal fat pad (BFP) flap, and a palatal flap.

In 1936, Rehrmann [11] described the use of a buccal advancement flap. Krompotie and Bagatin reported that the rotating gingivovestibular flap can also be applied for closure of an antrooral fistula. This technique is a modification of a vestibular flap with the aim of preventing the lowering of the vestibular sulcus, which regularly accompanies the application of vestibular flaps [12]. The flap with its simplicity, reliability, and versatility is the most commonly used method for OAF closure [5, 13].

In this technique, a broad-based trapezoid mucoperiosteal flap is created after excising the epithelialized margins and giving two vertical release incisions to develop a flap with adequate dimensions to be sutured over the defect. Its broad base enables a better blood supply to the flap. Flap coverage is improved by horizontal periosteal incisions. Falci et al. described a modification of this technique for OAF closure. The mucosal margins of the fistula were sutured together prior to the reflection of the buccal flap. Then, the buccal flap was pulled over this sutured site and tucked under the palatal flap, which was elevated simultaneously with the buccal flap [13]. Killey, in 1972, studied 362 cases using this technique. The results revealed success in 336 (93%) cases [5]. However, the potential disadvantage of using this flap for OAF closure is the reduction of buccal sulcus, which makes it difficult to use prosthesis in the future [1]. Other disadvantages include postoperative pain and swelling as a result of the reflection of a mucoperiosteal flap. Currently, a reduction in the buccal sulcus can be overcome by implant-retained overdentures.

Môczáir [14] described closing alveolar fistulas by the buccal sliding flap, shifting the flap one tooth distally. This technique produces only a negligible change in the depth of the buccal vestibule. A drawback of this approach is that it requires a large amount of dentogingival detachment in order to facilitate the shift, which may result in gingival recession and periodontal disease.

The first description of a technique for closing oroantral fistula using a full-thickness palatal flap based on greater palatine artery dates back to Ashley [15]. Advantages of the palatal flap include high vascularity, generous thickness, and quality of tissue. Moreover, this flap is more resilient, less prone to infection, very resistant to lacerations, and does not lead to lowering of the vestibule. However, the most significant disadvantages are flap necrosis [15], exposed bony surface, pain, and development of surface irregularities as a result of secondary epithelialization post operatively [2]. In 1980, Ehrl employed this technique with wide fistulas of 1 cm in diameter [16]. The technique consists of excising the epithelium from its edges and incising the palatal fibro-mucosa so as to create a flap with a posterior base, supplied by the greater palatine artery. The anterior extension of the flap must be wide enough to exceed the diameter of the bony defect and long enough to allow lateral rotation. Tension-free suturing should be performed [8].

The palatal flap has different forms that can be classified by thickness, namely, mucoperiosteal, or by the direction of movement, namely, straight advancement flap, rotation advancement flap, hinged flap, pedicled island flap, anteriorly based flap, submucosal connective tissue pedicle flap, and submucosal island flap.

The palatal mucoperiosteal rotation flap is particularly recommended for the late repair of oroantral fistula [8]. The base of the flap should be broad enough to cover the defect. A full-thickness palatal flap is typically performed lateral to vascular supply and 3 mm apical to the marginal gingiva of the teeth. The mucoperiosteal flap is raised from the anterior to posterior, rotated, and sutured to secure a tension-free closure of the fistula. [8]. With this technique, kinking formation at the rotation point and the base of the flap may compromise the vascular supply [8]. The most important advantages of the technique are rich vascularization, excellent thickness, and easy accessibility.

The palatal straight advancement flap is of limited use due to the inelastic nature of the palatal tissue, which reduces its lateral mobility. For the same reason, it is suitable for the closure of minor palatal or alveolar defects [17].

The palatal hinged flap has been used successfully to close small fistula of the hard palate, i.e., those less than 2 cm in diameter in a one-stage operation [18]. The procedure is based on raising a full-thickness flap directly adjacent to the fistula based along one fistula edge and turning this like a hinge over the fistula so that its buccal surface will lie uppermost in the fistula. The main advantage of this technique is that only a small raw area for granulation is left behind following closure of OAF.

Another single-stage local flap is the palatal pedicled island flap. It offers the great advantages of a rich vascular supply, adequate bulk, and mobility. In elevating the flap, incisions are performed according to the amount of donor tissue required to resurface the oral fistula. Incisions can be made at the junction of the hard and soft palate and within 5 mm of the dentition. This allows for harvesting a flap that can be rotated 180° without strangulation of the vascular pedicle. This procedure is the preferred flap for many surgeons because of its versatility, simplicity, and mobility [19]. The technique is ideal for the closure of posterior fistula due to its ability to transfer large well-vascularized area of tissue. Furthermore, the donor site defect, which overlies the hard palate, decreases the donor site morbidity. Therefore, use of this procedure is limited in closure of anterior defect as a result of stretching of these vessels when the flap is advanced too far anteriorly. The palatal anteriorly based flap is particularly useful for closure of large oroantral fistula and correction of defects at the tuberosity region. The procedure involves a lateral transposition of mucoperiosteum of the posterior third of the hard palate with an anteriorly based palatal flap. The flap is raised to bridge large defects without leaving any considerable exposed raw area [20]. The modified submucosal connective tissue flap was designed by Dergin et al. [21] for closure of OAF. Elasticity of the flap prevents folding formation and allows for better manipulation and adaptation in the closure of an OAF in the second and third molar region. Another advantage is that no palatal acrylic plate is required postoperatively. In this technique, an H-type window-like incision is made in the palatal mucosa 4 mm from the gingival margins of the posterior teeth, with the medial incision 2–3 mm from the midline. After excising the fistula wall and curetting the granulation tissue, the mucosa of the two minor flaps of the H-type window-like incision is raised and separated from the underlying connective tissue without jeopardizing the continuity of the mucosal flap. The underlying vascularized connective tissue is dissected in the premolar-canine region, where the incisive and greater palatine arteries anastomose. The connective tissue is raised with periosteum and rotated. The rotated flap is passed through a full-thickness tissue tunnel that had been previously prepared on the palatal side of the OAF. The flap is inserted under the buccal mucosa and sutured without any tension. The H-type minor flap is also sutured and left for primary healing. Hara and Ito designed the submucosal connective tissue pedicle flap by dividing the flap into an upper mucosal layer and underlying connective tissue layer to overcome the problem of bone exposure at the donor site [22]. The technique is achieved by separating the full-thickness palatal flap into a mucosal layer and an underlying connective tissue layer. The submucosal connective tissue flap is used to close the fistula, and the mucosal part of this flap is then returned to its original position and sutured in place to obtain primary closure. They reported that the healing at the donor site occurred within 1 month [22]. The disadvantages of this technique are the difficulty of the dissection and possibility of injuring the blood supply [22]. A submucosal island flap is indicated in the closure of a large oroantral fistula. With this technique, a palatal pedicle flap based on the greater palatine vessels is formed. Depending on the size, shape, and location of the fistula, the anterior part of the submucosal connective tissue flap is made. In addition, the anterior part of the submucosal connective tissue is used to design the island flap. After raising the palatal mucoperiosteum, the island flap is passed under the alveolar tissue bridge across the defect and sutured to the edge of the fistula without tension. The significant advantages of this flap are ample blood supply and mobility without tissue bunching [23]. In addition, healing is satisfactory without denuded bone. Yamazaki concluded that the dissection of the palatal flap into the epithelial layer and the underlying connective tissue does not pose technical difficulty [23]. A palatal pedicle flap is used to close an OAF with the advantages of preserving keratinized mucosa and buccal sulcus depth in the area of the fistula. The flap is a one-stage procedure. Preoperative procedure includes making a self-curing acrylic resin plate over the patient’s cast model. The first surgical step is excision of the epithelial fistula wall, followed by division of the flap on the palatal mucosa through an incision, superficially to the periosteum. The flap is passively positioned and thoroughly sutured. The donor site is then protected with surgical cement and held in place by the self-curing acrylic resin plate. The acrylic plate is fixed with bone screws and maintained in place for 10 days [24]. The ratio of length to thickness of the flap is critical for the survival of the palatal pedicle flap, thus splitting of the flap should be carried out very judiciously [24]. The Random palatal flap is considered an adequate option for closure of oroantral fistula in difficult locations such as in the tuberosity area. Lee et al. reported a success rate of 76% of random palatal flaps in 21 patients [25]. He pointed out that the most important factor determining the clinical outcome of palatal flaps was an appropriate length to width ratio. The random palatal flap is based on the anastomoses spread throughout the palate; all rely on greater palatine artery for nutrition. The buccal fat pad (BFP) is a lobulated mass of adipose tissue surrounded by a thin fibrous capsule, located between the buccinator muscle medially, the anterior margin of the masseter muscle, and the mandibular ramus and zygomatic arch laterally [26]. The buccal pad of fat is exposed through a 1 cm long vertical incision in the reflected periosteum posterior to the zygomatic buttress. Thereafter, it is gently manipulated by pressing extraorally below the zygomatic arc. Finally, the fat pad is sutured to the palatal tissue, covering the oroantral fistula [27]. The BFP derives its blood supply from branches of the superficial temporal, maxillary, and facial arteries. The advantages of this technique include good epithelialization of the uncovered fat and a high rate of success due to the BFP’s ample vascularity and proximity to the recipient site. Other beneficial features of the BFP flap are the straightforward harvest and minimal dissection required to harvest and to mobilize the flap [28]. The study of the long-term effectiveness of the BFP technique in the closure of large OAFs supported these features [29]. The disadvantage is the decrease in the vestibular height. It has been concluded that closure of large defects could involve complications such as graft necrosis or new fistulas [30]. The buccal fat pad is a feasible option for cases with damage to the alveolar buccal or palatal mucoperiosteum, cases that have failed with other methods, and repairs of large defects in the tuberosity area [31]. However, success of the buccal fat pad is extremely influenced by the communication size [32].

Free mucosal grafts (FMG) or connective tissue grafts (CTG) are suitable for the closure of small to moderate size defects in the premolar area as well as small to medium size-persistent defects. In contrast to the techniques described so far, the harvested grafts are not directly vascularized. The flap initially receives its nutrients within the *first three postoperative days* by diffusion alone, so-called plasmatic circulation. After day 3, the nutrition is provided by the ingress of blood capillaries from the recipient bed for revascularization. However, the size of the defect and the thickness of the graft play a crucial role in plasma circulation and nutrition of the transplanted cells. The main donor site for FMG/CTG is the palate, the maxillary tuberosity or edentulous alveolar ridges. Struder demonstrated that the thickest part of the palate and the most favorable donor site is in the premolar area [33]. The thickness of the mucosa decreases distally and becomes thicker once more in the area of the tuberosity. However, the inadequate area is small compared to the premolar area. To perform a FMG, the wound margins of the recipient bed are de-epithelialized according to the techniques already described and the defect to be closed is measured. Using a template, the size of the defect can be projected onto the palatal premolar area and the graft can be harvested. Within 3–4 weeks, the palatally exposed site heals completely above the free granulation. The donor site of the subepithelial connective tissue graft extends from the canine region to nearly the palatal root of the first molar. Free mucosal grafts (FMG) are suitable for closing the OAF to the maxillary second premolar region. Laterally, a distance of 2 mm from the gingival margin should be considered the minimum and a minimum 2-mm zone of the *marginal gingiva* should be maintained at the donor site. Medially, the boundary is the vascular-nerve bundles, which, depending on the anatomy of the palate, are 7, 12, or 17 mm away from the palatal cemento-enamel junction. After the incision, the subepithelial connective tissue is dissected and harvested according to the size of the defect. The harvested grafts (FMG/CTG) are then placed on the defect and adapted by sutures on the de-epithelialized wound margins. The pedunculated subepithelial connective tissue graft is particularly *well suited* for covering defects in the molar region. Similar to a non*-pedunculated* graft, this technique also uses a variety of incision techniques. In our polyclinic, we prefer to use the “trap door” technique favored by Wachtel [34]. According to Wachtel’s technique, an incision is performed at a distance of 2 mm from the gingival margin and extends, according to the extent of the defect, to the canine region. Subsequently, the scalpel is then angled off; a mucosal flap is prepared about 1.0–1.5 mm medially and a step approximately 1.5 mm is made. The subepithelial graft is then prepared mesially, medially, laterally, and basally. An *undermining* “*bridge*” between the alveolus or defect and the incision is prepared in the mucosa, and the graft is then placed under the bridge in the defect and fixed at the wound margins with sutures. As with the pedunculated palatal rotation flap, the blood supply to the graft is provided through the greater *palatine* artery, the anastomosis of the nasopalatine artery and sphenopalatine artery. In addition to the preservation of the vestibule and the associated favorable prosthetic options, the pedunculated subepithelial connective tissue graft is applicable to the majority of possible defects. Considering the blood supply by the greater *palatine* artery, clinicians and patients can expect a high probability of graft success. Due to its high elasticity, the graft can be effortlessly adapted free of tension [8]. With the use of this technique and the application of a step according to Wachtel, the wound margins can be optimally adapted and wound healing can be ensured by primary intention. Furthermore, there is no free exposed area so postoperative complaints are low [35]. The disadvantages of this technique include the long duration, high costs, and the ability of the *surgeon* [35]*.*

The tongue is an excellent donor site for soft tissue defects of the oral cavity, due to its pliability, position, and abundant vascularity. Tongue flaps can be created from the ventral, dorsal, or lateral part of the tongue [36]. The surgical design of the flap is dictated by the location of the defect. A lateral tongue flap has been described as a suitable method for the closure of large OAF [37]. The tongue flap has reported success rates varying from 85 to 95.5% [37]. Complications of the technique include hematoma formation that can compress the pedicle leading to necrosis of the flap, wound dehiscence, and temporary loss of tongue sensation and taste [38]. Additional disadvantages are the requirements for general anesthesia and multiple operations. The temporalis muscle flap is another distant flap, which can be used for closure of orofacial region defects. It has been indicated for one-stage closure of large oroantral communications. The temporalis fascia is sectioned above the arch to permit flap rotation. It is then brought into the oroantral fistula through a tunnel created in the infra temporal fossa. The temporalis muscle flap is far less bulky, well-vascularized, and more pliable; with minimal functional and esthetic sequelae; and in closer proximity to the oral cavity [39].

The use of bone autografts for closing OAFs has been recommended in the literature [40]. This procedure is indicated in closure of defects larger than 10 mm or in the case of failure of conservative methods to close the defect [41]. Autografts harvested from the extraction socket, retromolar area, zygomatic process, chin, or distant sites like the iliac crest have been used for repairing the bony defect in maxilla [9, 42]. Harvesting bone from intraoral donor areas offer the advantage of reducing the demands made on patients postoperatively.

Closure of OAF with a bone graft harvested from the iliac crest should be indicated for large defects because of the significant inherent donor site morbidity, prolonged postoperative pain, and possible sensory disturbance [40, 43]. The use of monocortical bone grafts harvested at a chin site block for closure of an OAF is recommended for patients affected by maxillary atrophy requiring sinus augmentation before implant placement [9]. A monocortical block graft is harvested at the chin by using a trephine with an inner diameter matching the size of the round bony defect. The graft is then press-fit into the defect. Soft tissue closure is established by using a flap.

A retromolar bone graft is a viable procedure for OAF closure. However, harvesting of a retromolar bone can occasionally be combined with removal of the third molar, which may affect acceptance of the procedure by patients [44]. When compared to chin bone grafts, the significant disadvantage of the retromolar donor area is the confined amount of bone available [45]. The incision is made medial to the external oblique ridge in an anterior direction and terminated in the first molar area to avoid interference with the mental nerve branches. A mucoperiosteal flap is elevated, and the exposed bone area is evaluated in consideration of the amount of bone needed at the defect site. A microreciprocating saw is used to make the osteotomies. The bone block is carefully lifted to ensure that the inferior alveolar nerve is not trapped within the graft. Osseous irregularities are trimmed with chisels or by using a large bur. The flap is repositioned and sutured [45].

Zygomatic bone is a suitable donor site for OAF closure. The technique is indicated when a modest amount of bone is needed [46]. In this procedure, an incision is made through the alveolar mucosa about 5 mm above the mucogingival junction, starting between the first and second molars, and proceeds anteriorly to the first premolar area. A full-thickness flap is raised with a periosteal elevator. The dissection extends to the inferior aspect of the infraorbital nerve and around the inferior half of the body of the zygoma. The lateral border of the maxillary sinus is visualized, and the inferior border of the orbital rim is palpated. Bone harvesting is started just above the inferior border of the zygomatic rim and lateral from the maxillary sinus. The incision is closed with running or interrupted resorbable sutures [46].

This method offers the advantage of proximity of the donor area to the recipient area, which minimizes surgical time and patient discomfort [46]. Moreover, surgical postoperative complications after zygomatic bone harvesting are minimal.

Recently, auricular cartilage graft has been used for the closure of OAFs. A full-thickness flap is raised at the defect site [47]. A semicircular incision is then made posteriorly over the conchal cartilage. The conchal cartilage with overlying perichondrium is exposed with a blunt dissection. The harvested auricular graft is then adapted on the defect site and sutured with the surrounding tissue. The mucoperiosteal flap is then advanced and sutured with the palatal tissue. The technique is biocompatible, highly resistant to infection, and easy to harvest. Additionally, it does not require vascularization for the integration to the recipient site. Consequently, there is a decrease in the failure rate of the graft [47]. A disadvantage of this procedure is the potential formation of a defect at the donor site.

The use of septal cartilage especially for larger oroantral fistulas was documented [48]. A buccal mucoperichondrial flap is raised, generally starting two teeth mesial and ending, and if feasible, one tooth distal to the site of the fistula. An incision is performed at the caudal end of the septal cartilage, and a mucoperichondrial flap is raised on one side. A cartilage island is outlined and dissected free. The cartilage is trimmed and insinuated into defect as a horizontal plate [48]. Saleh et al. obtained a success rate of 95.7% in their study using septal cartilage [48].

According to Aladag et al., the modified Caldwell-Luc Approach is a satisfactory method to close oroantral defects [49]. In the technique, which includes endoscopic examination using the Caldwell-Luc approach, the inside of the maxillary sinus is explored fully. The bone graft can be harvested from the bone of the anterior wall of the maxillary sinus by accessing the surgical entry tract. The positive features of the technique include the use of autogenous grafts, easy and adequate harvesting of the graft along the surgical route, and no need for a flap. Among its disadvantages are the fact that it requires endoscopic surgical equipment and experience.

An autogenous bone graft and platelet-rich fibrin (PRF) membrane as a treatment strategy for closure of OAF has also been proposed [50]. PRF is a product of centrifuged blood. The biochemical components of PRF are well-known as factors acting synergistically in the healing process. This includes platelet-derived growth factor (PDGF), whose components are the reason why PRF has anti-inflammatory properties. The PRF membrane covers the graft, while the components contained in it have positive impact on its integration. A trapezoidal mucoperiosteal flap is formed in the oral cavity vestibule. The alveolar width of alveolus and the average height of alveolar bone lamina from the side of the oral cavity vestibule and from the side of the palate are measured intraoperatively. The next stage depends on the cavity diameter and involves collection of monocortical bone blocks from the mental protuberance or mandible oblique line. The bone blocks are formed in a way which makes it possible to wedge them in the cavity and tightly close the defect. The graft is stabilized using a bicortical screw or a titanium mini-plate. Bone irregularities are smoothed. The graft and surrounding bone are covered with a PRF membrane. Thereafter, the membrane is tightly sutured without tension the vestibule flap [50].

More recently, triple-layered was introduced by George [51]. This novel technique uses leucocytes-platelet-rich fibrin (L-PRF) membrane concomitantly with the buccal advancement flap and buccal fat pad. The platelet-rich fibrin membrane is placed over the buccal fat pad and completely covered by a buccal advancement flap. The positive feature of the L-PRF membrane is expediting the healing process by producing growth factors and leucocytes.

The use of PRF alone as a clot and a membrane for the closure of OAFs was documented by Assad et al. [52]. They advocated closing OAFs by using PRF individually. PRF was prepared by taking blood samples into glass-coated plastic tubes without anticoagulant. The samples were centrifuged immediately. A fibrin clot was formed in the middle part of the tube. Then, it was separated from other acellular plasma and red blood cells. Thereafter one third of the fibrin was cut off and inserted gently into the OAF. The remaining two thirds of the clot were pressed gently with sterile dry gauze to drive out the fluids and form the membrane. The OAF site was covered with the membrane which was sutured to the gingival margins. PRF can be considered an autologous biomaterial and as well as a membrane. PRF as a membrane and grafting material facilitates formation of mineralized tissue due to osteoconductive and/or osteoinductive properties possibly inherent in PRF.

### Allogenous materials

Multiple techniques have been described for the closure of OAFs using lyophilized fibrin glue of human origin [53]. In this technique, the fibrin glue is prepared and injected into the socket, together with the collagen sheet. Stajčić et al. stressed the importance of inserting the syringe above the floor of the antrum to protect the clot from airflow [53]. The technique is simple with few postoperative complaints. Importantly, there is no need to raise flaps; hence, the intraoral anatomy remains intact [54]. According to the manufacturer, the major disadvantages of the procedure are the risk of transmitting viral hepatitis and the preparation time required for the fibrin glue [53].

The use of lyophilized dura for closure of OAF was reported by Kinner and Frenkel [55]. In this technique, the sterilized dura is placed in a saline solution to regain its pliability. Thereafter, it is cut to size to make it cover the bony margins of the defect. Sutures are placed at each *corner* of the graft and then it is covered with a plastic plate for protection. The dura is exfoliated after 2 weeks. The simplicity of the technique and *non-surgical approach make it an* attractive option for OAF closures. However, the risk of transmitting pathogens is a main disadvantage.

### Xenografts

Lyophilized porcine dermis for closure of OAFs has been described in the literature [56, 57]. The technique reported good results when the porcine graft was either exposed to the oral environment or covered with buccal and palatal sliding flaps [56, 57]. According to Mitchell and Lamb covering the graft by buccal and palatal flaps is not necessary [56]. The main advantage of the collagen is potential incorporation into the granulation tissue, and thus, no need to remove it prior to complete healing [56].

Ogunsalu achieved both bony (hard tissue) and soft tissue using Bio-Gide® (porcine collagen membrane) (Geistlich Biomaterials, Wolhusen, Switzerland) and Bio-Oss® (non-sintered bovine bone materials) (Geistlich Biomaterials, Wolhusen, Switzerland) to close OAFs [58]. In this technique, the Bio-Oss® granules were sandwiched between two sheaths of a Bio-Gide® membrane for the hard tissue closure of oroantral defect. Thereafter, a full-thickness mucoperiosteal flap was raised and the Bio-Oss®–Bio-Gide® sandwich placed underneath. Then, the flap was repositioned, resulting in primary closure. There was an excellent bony regeneration which allowed placement of an endosseous implant. Radiographically, bony healing of the defect was observed after 8 months. The technique offers the unique advantage that no donor site surgery is necessary. The disadvantage in this technique is the need for a mucoperiosteal flap to cover the sandwich.

### Synthetic closure of OAFs

Various synthetic materials have been used for OAF closures. Use of gold foil and gold plate for the closure of OAFs was reported for the first time by Goldman and Salman, respectively [59, 60]. It is a simplified technique for the closure of oroantral fistulas. The technique consists of elevating the mucoperiosteum to expose the bony margins of the fistula. Then, the opening is covered with an overlapping margin of burnished gold foil.

The mucoperiosteal flaps are sutured across the gold foil without attempting to realize primary closure. Gold foil acts as a bridge for overgrowing sinus mucosa. After 6 weeks, the foil is exfoliated. A *disadvantage* of this *technique* is that it is *rather costly and the complete closure and healing requires a long period* [57]*.*

Aluminum plates were suggested for OAF closures [61]. According to Steiner, 36-gauge pure aluminum plate is used as a protective plate to aid in closure, using the same technique as in the gold procedure. Buccal and palatal tissues are approximated by sutures. Accordingly, the aluminum plate is constantly visible. After several weeks, the aluminum plate is removed from its initial position as a result of formation reparative tissue underneath. In addition to malleability and smoothness features, aluminum is inexpensive.

Tantalum is a highly biocompatible metal. It has been used for closures of OAFs by McClung and Chipps [62]. Along the same lines as the gold technique, tantalum foil is used as a protective plate to aid in closure. The Tantalum foil was removed after 9 weeks. They reported formation of granulation tissue following the closure.

Closure of OAFs by titanium plate with transalveolar wiring fixation was documented [63]. The results revealed good bony and soft tissue healing. Further, the use of two different materials titanium plates and stainless steel wires did not result complication or distaste because of galvanic current.

Polymethylmethacrylate has been introduced as an alternative technique for closing OAFs [64]. After 24 h of immersion in a sterilizing solution, the polymethylmethacrylate plate is placed over the defect. Mucoperiosteal flaps are then replaced without attempting to cover the acrylic plate. The polymethylmethacrylate plate is removed as soon as the edges become exposed. One of the common disadvantages of this technique is the required time for preparation.

Recently, a dual otorhinolaryngological/oral approach was described in a patient with an OAF complicated by maxillary sinusitis [65]. The investigators used the functional endoscopic sinus surgery (FESS) technique in combination with a titanium mesh to obtain optimal reconstruction and stabilization of soft tissue. A full-thickness vestibular flap was elevated, and the titanium mesh was fixed on the defect. Mesh removal was conducted after 6 to 18 months of healing based on clinic and radiographic evidence of OAF closure. The main disadvantage of this technique is the second surgery needed to remove the mesh. Despite this drawback, use of a titanium mesh assures predictable healing, mechanic scaffolding, and tissue stability.

Zide and Karas used nonporous blocks of hydroxylapatite to close oroantral fistulas by carving the blocks to fit the bony defect and encircling them with a wire for stability when needed [66]. The investigators reported natural extrusion of the blocks without recurrence of a fistula [10]. The technique offers a number of advantages, including ability to have a press-fit graft closure and no morbidity associated with a second-site surgery.

Various sizes of hydroxylapatite implants have also been used to close oroantral fistulas [67]. Further, the remaining space in the socket was filled by hydroxylapatite granules. Oral mucosa was approximated without complete closure [10]. Considering extrusion, the technique resulted in no cases of hydroxylapatite implant extrusion. Disadvantages include high costs and the need for various implant sizes to allow for size selection.

The use of a bioabsorbable root analog made of β-tricalcium phosphate for closure of oroantral fistulas was proposed by Thoma et al. [68]. The root replicas were fabricated chair side, using a mold of the extracted tooth [10]. The investigators reported that the healing was uneventful. However, fragmentary roots or overly large defects prevent replica fabrication or accurate fitting of the analog. The technique is simple and fast.

### Other techniques

Kitagawa et al. advocated third molar transplantation as a suitable option for closure of OAFs [69]. The investigators successfully closed two cases of OAFs by immediate upper and lower third molar transplantation.

The donor teeth were carefully extracted and transplanted to the prepared recipient bed. Firm finger pressure and light tapping provided good stabilization of the tooth on the recipient bed and produced a complete simultaneous closure of the OAF. Endodontic treatment was carried out after 3 weeks. The researchers reported that third molar transplantation was a simple and excellent treatment option to close small OAFs. However, third molar transplantations have some drawbacks: the requirement of a sufficiently developed third molar of an appropriate shape and size, and the risks of ankylosis and root resorption if not carried out with proper technique.

Hori et al. proposed interseptal alveolotomy as one of possibilities for closure of OAFs [70]. The technique, based on the Dean preprosthetic technique, is used for the purpose of smoothing the alveolar ridge. The extended Dean technique is performed in such a way that the interseptal bone is removed, followed by the fracturing of the buccal cortex in the direction of the palate. Sutures are used for soft tissue closure. The technique offers the advantage of facilitating spontaneous postoperative healing with less postoperative swelling, supported by the bony base. The most important advantage of this technique, compared with the buccal flap technique, is that it assures closure of soft tissue without creating tension. However, limitations of this method are that it requires both a space of less than 1 cm between the adjacent teeth and adequate alveolar ridge. Moreover, there is a risk of inflammation as a result of the required buccal bone fractures due to formation of bone sequesters and possible imperfect soft tissue closure in the case of an incomplete fracture.

Use of guided tissue regeneration has been documented by Waldrop and Semba [71]. This method uses an absorbable gelatin membrane, allogenic bone graft material, and a nonresorbable expanded polytetrafluoroethylene (ePTFE) membrane. After flap reflection, an absorbable gelatin membrane is placed over the OAF with its edges on the bony margins of the perforation, which serve as a barrier for the bone graft material and prevent displacement of the graft material into the antrum and sinus epithelial cell migration. A layer of allogenic bone graft material is put on the membrane. The nonresorbable ePTFE membrane is used to cover the bone graft material, and the soft tissue flap is placed over the membrane. This membrane promotes selective cell population with subsequent regeneration. Eight weeks after insertion, the barrier membrane is removed. After removal of the inner aspect of the flap adjacent to the ePTFE membrane, the mucoperiosteal flap is replaced. Closure of the OAF was clinically confirmed by bone formation, although this was not confirmed histologically. One of the *disadvantages* of this technique is the need for an additional surgery to remove the nonresorbable ePTFE membrane. A further disadvantage is the need for a full-thickness flap. Götzfried and Kaduk developed an alternative procedure to close OAFs without surgical intervention [72]. According to the investigators, prolamin occlusion gel is directly injected into the perforation and hardens within a few minutes to form a barrier. One week later, granulation tissue is formed and the prolamin gel completely dissolves after 2 to 3 weeks [72]. This technique proved to be well tolerated by patients and results in fewer postoperative complaints compared with other procedures [68]. The disadvantage of this technique is chiefly its high material cost. Additionally, the technique is less appropriate for closure of OAFs greater than 3 mm [68].

Biostimulation with laser light for closure of OAFs was suggested by Grzesiak-Janas and Janas [73]. In this method, 61 patients were subjected to 3 cycles of extraoral and intraoral irradiation with a CTL 1106 biostimulative laser of 30-mW power with a tip-emitting light of 830-nm wavelength for 10.5 min and for four consecutive days. The researchers demonstrated a complete closure of OAFs. This technique eliminates the need for a surgical procedure. The technique has the disadvantage of being expensive and requires many visits to accomplish complete closure.

Logan and Coates described a procedure that provided closure of OAF in immunocompromised patients [74].

The oroantral fistula was de-epithelialized under local anesthesia, and the patient wore an acrylic surgical splint continuously for an 8-week period. The acrylic surgical splint covered the fistula and the edentulous area including the hard palate. The investigators reported complete healing of the oroantral fistula after 8 weeks. The technique is considered a very useful option when a surgical intervention is contraindicated because of immunosuppression.

## Discussion

A comprehensive review of available surgical and non-surgical methods for closure of OAFs has been described. The databases were selected to be comprehensive and to cover a broad range of methods for closure of OAF. A limitation of this review is the fact that reporting of new techniques for closure of OAF was mostly in case reports. Therefore, randomized controlled clinical trials were not implemented to assess the quality and feasibility of the new treatment strategy. Accordingly, further research is recommended to investigate the success of varied techniques. It is recommended to investigate the condition of antral mucosa. *Successful closure* of the oroantral fistula should be preceded by the complete elimination of any sinus pathology [17]. Additional surgery may be required to remove the diseased lining if severe sinusitis is present prior to antrum exposure [75]. Considering the different interventions available to close OAFs, it is important to identify the best intervention techniques to help clinicians to manage patients with OAF efficiently. The choice of the procedure is controversial. Most oral and maxillofacial surgeons prefer either buccal or palatal flaps in case primary suturing of the soft tissue cannot adequately close the oroantral fistula. Others claim that palatal flaps are preferable because of their ample blood supply. A review of literature on advantages of the palatal flap revealed features of abundant vascularity, satisfactory thickness, and resistance to laceration. Recently, because of the continued need for implant rehabilitation, bony closure of OAFs is increasingly being employed in the closure of oroantral fistula. Closure of OAFs with bone grafts harvested from intraoral donor sites paves the way for implant surgery in terms of sinus lifting. However, autogenous bone grafting has some negative aspects including the necessity for a second surgical procedure for bone harvesting and concerns about donor site morbidity. Therefore, other grafting materials have been investigated for closure of OAFs [76]. It is quite evident that the mechanical properties, biological behaviors and biodegradation mechanisms vary for different graft materials. Unlike *allografts* or *xenografts*, by nature, *alloplastic* or *synthetic materials* limit the *risk* of cross infection transmission of pathogens. Alloplastic materials are easy and simple; however, their disadvantages include being time consuming and high cost and requiring exfoliation, which may limit their use as an alternative surgical technique for closure of OAF. Another consideration is that, the alloplastic material procedures do not influence the buccal vestibular depth. Moreover, the use of alloplastic materials would result in an area free of raw for granulation following closure of the defect. Recently, the use of platelet-rich fibrin for closure of OAF is simple, inexpensive, and may be an effective method for closing OAF. However, it needs more evidence-based data. In view of the foregoing discussion, this literature review provides an overall general theoretical point of view to make sense out of available techniques for closure of OAFs.

## Summary and conclusion

By reviewing the literature, we can conclude that in selecting the surgical approach to close an oroantral fistula, different parameters have to be taken into account, including location and size of fistula as well as its relationship to the adjacent teeth, height of the alveolar ridge, persistence, sinus inflammation and the general health of the patient.

A small oroantral fistula of less than 5 mm in diameter can be *closed* immediately and effectively by *suturing the gingiva* with a *figure eight suture*. *If this does not provide adequate closure*, *a flap procedure is indicated*. The closure of an oroantral fistula can be performed by different techniques. Buccal flaps are often indicated in closure of small to moderate size defects. However, reduction of buccal vestibular height like the Rehrmann flap following this procedure makes it difficult to use prosthesis in future. Alternatively, the buccal advancement flap, and harvesting retromolar bone, prolamin gel, acrylic splint, guided tissue regeneration (GTR), and bone grafts can be successfully used for closures of OAF of less than 5 mm. However, over recent years grafts including free mucosal (FMG) or connective tissue grafts (CTG) are increasingly being used to close small to moderate size defects in the premolar area. The combination of the buccal flap and the buccal fat pad is appropriate for fistulae located in the second and third molar area. Closure of defects larger than 5 mm can be performed using one of the following procedures: combination of an endoscopic and per-oral BFP flap approach, BFP flap, pedicled buccal fat pad, modified submucosal connective tissue flap, distant flaps, autogenous bone grafts, allogenous, synthetic materials**/**metals, and other techniques. Ideally, the Bio-Gide®-Bio-Oss®^−^ Sandwich technique (Geistlich Biomaterials, Wolhusen, Switzerland) achieves both bony and soft tissue closure of OAF. In any case, it should be clear here that the technique for closure the OAF always depends on the indication and as well as the experience of the surgeon. It is often easier for a beginner to use a PTFE membrane or Rehrmann flap than to mobilize elaborate flap techniques and structures which are at risk, e.g., bone graft transplantation (BGT), injury of the arteria palatina and corpus adiposum buccae (buccal fat pad). Moreover, when searching deeply for the nervus faciali, injury of the nerve may occur. The dentist must be able to assess his abilities and, accordingly, choose the therapy to close the OAF.

## Abbreviations

BFP: Buccal fat pad
BGT: Bone graft transplantation
CTG: Connective tissue grafts
FMG: Free mucosal graft
GTR: Guided tissue regeneration
OAF: Oroantral fistula
PRF: Platelet-rich fibrin
